# Capsaicin Attenuates Lipopolysaccharide-Induced Inflammation and Barrier Dysfunction in Intestinal Porcine Epithelial Cell Line-J2

**DOI:** 10.3389/fphys.2021.715469

**Published:** 2021-09-24

**Authors:** Xiaoya Zhao, Bingqi Dong, Marissa Friesen, Shangxi Liu, Changqing Zhu, Chengbo Yang

**Affiliations:** ^1^Department of Animal Science, University of Manitoba, Winnipeg, MB, Canada; ^2^School of Food Science, Nanjing Xiaozhuang University, Nanjing, China

**Keywords:** capsaicin, LPS induction, IPEC-J2, inflammation, TLR4/NF-κB pathway, gut integrity

## Abstract

Capsaicin is a spicy, highly pungent, colorless, vanilloid compound found in chili peppers with anti-inflammatory, antioxidant, anti-cancer, and analgesic properties. However, the protective effects of capsaicin on the pig intestine during inflammation are yet to be explored. This study investigated the effects of capsaicin on the gut inflammatory response, intestinal epithelial integrity, and gene expression level of nutrient transporters in a model of lipopolysaccharide (LPS)-induced inflammation in non-differentiated intestinal porcine epithelial cell line-J2 (IPEC-J2). The results showed that the pre-treatment of cells with capsaicin (100 μM) significantly decreased the gene expression and secretion of proinflammatory cytokines induced by LPS through Toll-like receptor 4 (TLR4)/NF-κB signaling pathway. In addition, pre-treatment of cells with capsaicin also increased both gene and protein abundance of tight junction proteins. Furthermore, pre-treatment cells with capsaicin significantly increased trans-epithelial electrical resistance (TEER) and decreased permeability of fluorescein isothiocyanate-dextran (FD4) from the apical side to the basolateral side compared with the control (*P* < 0.05). Additionally, pre-treatment of cells with capsaicin upregulated the mRNA abundance of nutrients transporters such as Na^+^/glucose cotransporter 1 (SGLT1). These results suggested that capsaicin could attenuate LPS-induced inflammation response through TLR4/NF-κB pathway and improve barrier integrity and glucose absorption.

## Introduction

Presently, enteric diseases are a significant cause of morbidity and mortality in the swine industry, causing economic loss and reduced growth and production efficiency (Moxley and Duhamel, 1999). There is a wide variety of enteric diseases threatening the health of swine worldwide, and each disease is selective to a specific class of pigs. For example, *Escherichia coli* favors suckers and weaners and can symptomatically appear as diarrhea which affects the growth and health of the piglets and can be a possibly life-threatening disease (Rhouma et al., 2017). Furthermore, since enteric diseases can easily induce an inflammatory response, damage the intestinal morphology, and resulted in a defective intestinal epithelial barrier, it is necessary to prevent intestinal inflammation and dysfunction by maintaining an optimally functioning gastrointestinal tract.

Broad-spectrum antibiotic use in livestock has been shown to increase growth performance and treat and prevent enteric disease. However, antibiotic resistance in livestock and humans is an increasingly urgent problem in public health (Adjiri-Awere and Van Lunen, 2005). Many countries have already banned antibiotics as growth promoters in swine and poultry due to the risks such as multidrug-resistant pathogens being transferred to humans and pig meat containing antibiotic residues. Therefore, it is a matter of urgency to develop antibiotic alternatives.

Recent studies have investigated the use of phytochemicals as antibiotic alternatives in livestock production because phytochemicals have potential anti-inflammatory effects that could attenuate chronic inflammation and prevent chronic diseases (Mroz, 2005; Omonijo et al., 2018). Capsaicin is the phytochemical present in chili peppers responsible for the spicy sensation during consumption, and in its pure form, it is hydrophobic, colorless, and highly pungent (McCarty et al., 2015). Capsaicin belongs to the vanilloid family and binds to the transient receptor potential vanilloid 1, which involves sensations such as pain and heat and locates in sensory neurons and other places like the gut epithelial cells. Capsaicin is currently used in topical treatments because it produces analgesic effects by depleting neurotransmitter substance P responsible for transmitting pain signals to the brain. In addition, capsaicin possesses anti-obesity abilities by lowering chronic low-grade inflammation associated with obesity (Chang et al., 2011). In particular, researchers reported that capsaicin supplementation could improve the gut barrier abnormalities and lower gut permeability by reducing the expression of pro-inflammatory cytokines and enhancing the abundance of short chain-fatty acids producing bacteria, which finally prevent obesity (Kang et al., 2017). That implies the capsaicin could potentially have a protective effect on the prevention of intestinal inflammation. It also has the potential to improve gut health by enhancing the gut barrier and intestinal microbiota.

Lipopolysaccharide (LPS) is a component of the cell wall in gram-negative bacteria, and it is commonly used as a model to study the inflammatory response (Park and Lee, 2013). TLR4 is a member of the TLR (Toll-like receptor) family, which acts as the major LPS receptor (Park and Lee, 2013). Studies have demonstrated that the binding of LPS by TLR4 and its co-receptors myeloid differentiation protein-2 (MD-2) could lead to the TLR4–MD-2 complex dimerization, subsequently contributing to the recruitment of myeloid differentiation factor 88 (MyD88) (Zusso et al., 2019). This protein-protein interaction cascade could initiate infection through the activation of the nuclear factor kappa-light-chain-enhancer of activated B cells (NF-κB), activating NF-κB which controls the transcription of pro-inflammatory cytokines such as interleukin-1beta (IL-1β), IL-8, and tumor necrosis factor-alpha (TNF-α), as well as anti-inflammatory cytokines like IL-10 (Ngkelo et al., 2012). Those pro-inflammatory cytokines could interact with immune cells and cause an acute inflammatory response, while IL-10 could inhibit pro-inflammatory cytokine production. Previous studies reported that the overexpression of pro-inflammatory cytokines such as TNF-α, IL-1β, and IL-6 could contribute to destructive cell damage to the intestinal epithelial monolayers and then damage the intestinal barrier (Kanwar and Kanwar, 2009). The intestinal barrier, primarily formed by epithelial cells, provides protection and selective permeability to regulate the passage of various molecules through the intestinal wall (Turner, 2006). The apical surface of the intestinal epithelium functions to absorb, and the basolateral surface specializes in nutrient transport from the cell to the bloodstream (Kong et al., 2018). The gastrointestinal epithelium can also prevent the passage of pathogens and toxins into the systemic circulation, which plays a vital role in preventing enteric disease (Pluske et al., 2018). Barrier function in the gastrointestinal tract is maintained through tight junctions, located in the paracellular space between adjacent epithelial cells, composed of crucial tight junction proteins (Turner, 2006). The permeability of the tight junction determines the barrier’s efficiency. Therefore, when pathogens or toxins invade, causing inflammation and eventual cell death, the barrier’s integrity may be compromised due to damages tight junctions (Turner, 2006; Yan and Ajuwon, 2017). Therefore, tight junctions are widely considered as an effective therapeutic target for maintaining gut barrier function and preventing intestinal inflammation.

So far, the mechanism of capsaicin’s anti-inflammatory properties and ability to regulate the barrier function in the non-differentiated swine intestine epithelial cells (IPEC-J2 cells) when stimulated by LPS has not been sufficiently studied. Therefore, we want to examine how capsaicin regulates the gut barrier function and inflammatory response in the IPEC-J2 cell line when stimulated by LPS. We hypothesis that capsaicin will maintain the barrier function by regulating the expression of inflammatory cytokines and the expression of nutrients transporters and tight junction proteins, eventually attenuating the LPS-induced inflammatory response.

## Materials and Methods

### Materials and Reagents

Capsaicin (Sigma-Aldrich, Oakville, Ontario, Canada), fluorescein isothiocyanate-dextran (FITC-dextran, 4 kDa), ethanol, and LPS (Sigma-Aldrich).

### Cell Culture and Treatments

IPEC-J2 cell line (ACC 701, RRID:CVCL_2246) was obtained from the DSMZ-German Collection of Microorganisms. IPEC-J2 cells were maintained in a 75 cm^2^ flask in DMEM/Ham’s F-12 (1:1) (*Invitrogen*, Fisher Scientific, Ottawa, Ontario, Canada) supplemented with 10% fetal bovine serum (FBS) (Hyclone, Canadian origin; Fisher Scientific, Ottawa, Ontario, Canada), penicillin (100 IU/mL), streptomycin (100 μg/mL) at 37°C in a humidified atmosphere of 5% CO_2_ and 95% O_2_. Culture medium was replaced every two to three days. When reaching 80 to 90% confluency, cells were split into 96, 6 well plate or 24 well transwell inserts (Corning Costar, New York City, New York, USA) respectively for different experiments as described in the following sections. For capsaicin treatment, 100 mM stock solution was freshly prepared in 95% ethanol and diluted in complete medium at appropriate concentrations. An equal level of ethanol was added to all the experimental groups to eliminate the effects of ethanol.

### Cell Viability Assay

Cell viability assay was conducted by using the water-soluble tetrazolium salts (WST-1) cell proliferation reagent (Sigma-Aldrich), following the manufacturer’s manuals. Briefly, IPEC-J2 cells were cultured in 96 well plates for two weeks and then incubated with capsaicin at concentrations of 0, 50, 100, 150, 200, and 300 μM, respectively, for 24 h. The cells were then washed twice with PBS, and a 100 μL fresh culture medium containing 10% WTS-1 was added to each well. After 1 h of incubation, the absorbance at 450 nm was measured using a Synergy H4 hybrid multi-mode microplate reader (BioTek, Winooski, VT). Cell viability data were presented as a percentage of untreated control cells.

### Cytokines Measurement by ELISA

IPEC-J2 cells were cultured in 6 well plates and were initially pre-treated with capsaicin (100 uM) for 2 h and then stimulated with LPS (10 μg/mL) for 6 h. TNF-α and IL-8 in the supernatants were measured using porcine ELISA kits according to the manufacturer’s instructions (Thermo Scientific). Briefly, 100 μL of culture supernatant was used for both TNF-α and IL-8 assay and the absorption at 450 nm was read using a Synergy H4 hybrid multi-mode microplate reader (BioTek). Cytokines’ concentration was calculated according to a standard curve generated using seven 2-fold dilutions of TNF-α and IL-8.

### RNA Extraction and Real-Time PCR

IPEC-J2 cells cultured in 6 well plate were first pre-treated with capsaicin (100 uM) for 2 h and then stimulated with LPS (10 μg/mL) for 2 h. Total RNA was isolated using Trizol® reagent (*Invitrogen*) following the manufacturer’s instructions. RNA concentration and purity were determined by Nanodrop-2000 spectrophotometer (Thermo Scientific, Ottawa, ON, Canada) and the OD260:OD280 ratios of all RNA samples were between 1.9 and 2.1. Reverse transcription was performed with 1 μg of total RNA using the iScript^TM^ cDNA Synthesis kit (Bio-Rad, Mississauga, ON, Canada) to synthesize the first strand cDNA. Real-time PCR was carried out using SYBR Green Supermix (Bio-Rad) on a CFX Connect real-time PCR detection system (Bio-Rad). The primers for real-time PCR analysis were shown in Table 1 and the thermal profile for all reactions followed the previous study: 3 min at 95°C, then 40 cycles of 20 s at 95°C, 30 s at 60°C, and 30 s at 72°C. At the end of each cycle, the fluorescence was monitored for 10 s. To ensure amplicon specificity, each reaction was completed with a melting curve analysis (Omonijo et al., 2019). Real-time PCR data were analyzed using the 2^−ΔΔCT^ method to calculate the relative fold-change of target gene, and cyclophilin-A (CycA) acted as the reference gene (Livak and Schmittgen, 2001; Paszti-Gere et al., 2014; Farkas et al., 2015). Real-time PCR efficiencies were acquired by amplification of the dilution series of DNase-treated RNA according to the following equation: *E* = 10^(−1/slope)^ (Pfaffl, 2001). The expressed real-time PCR efficiency of all primers used in this project was between 96 and 105%. The R^2^ value was more than 0.99 for all reactions.

**Table 1:** Primers sequences used in the current study.

**Genes**	**Primer sequences (5^′^-3^′^)**	**Product Size (bp)**	**GenBank Accession**
TNF-α	F: TTCCAGCTGGCCCCTTGAGC	146	NM_214022.1
	R: GAGGGCATTGGCATACCCAC		
IL-8	F: AGAGGTCTGCCTGGACCCCA	126	NM_213867.1
	R: GGGAGCCACGGAGAATGGGT		
IL-1β	F: ACATGCTGAAGGCTCTCCAC	170	NM_214055.1
	R: CAGGGTGGGCGTGTTATCTT		
IL-10	F: AGCTGCATCCACTTCCCAAC	102	NM_214041.1
	R: GCCCATCTGGTCCTTCGTTT		
TLR4	F: GCCATCGCTGCTAACATCATC	108	NM_001113039
	R: CTCATACTCAAAGATACACCATCGG		
MyD88	F: CCATTCGAGATGACCCCCTG	183	NM_001099923.1
	R: TAGCAATGGACCAGACGCAG		
IRAK1	F: CAAGGCAGGTCAGGTTTCGT	115	XM_003135490.4
	R: TTCGTGGGGCGTGTAGTGT		
TRAF6	F: CAAGAGAATACCCAGTCGCACA	122	NM_001105286.1
	R: ATCCGAGACAAAGGGGAAGAA		
TAK1	F: GCCACCGTAAAACTGCTTCAT	196	NM_001114280.1
	R: GCTGGCTTTTCTGAGGTTGG		
NF-κB p65	F: GTGTGTAAAGAAGCGGGACCT	139	NM_001114281.1
	R: CACTGTCACCTGGAAGCAGAG		
ZO-1	F: GATCCTGACCCGGTGTCTGA	200	XM_021098896.1
	R: TTGGTGGGTTTGGTGGGTT		
OCLN	F: GAGAGAGTGGACAGCCCCAT	163	NM_001163647.2
	R: TGCTGCTGTAATGAGGCTGC		
CLDN3	F: CTACGACCGCAAGGACTACG	123	NM_001160075.1
	R: TAGCATCTGGGTGGACTGGT		
SGLT1	F: GGCTGGACGAAGTATGGTGT	153	NM_001164021.1
	R: ACAACCACCCAAATCAGAGC		
EAAC1	F: GTTCCTGATTGCCGGGAAGA	165	NM_001164649.1
	R: ATGGCGAATCGGAAAGGGTT		
ASCT2	F: GCCAGCAAGATTGTGGAGAT	206	XM_003355984.4
	R: GAGCTGGATGAGGTTCCAAA		
B^0^AT1	F: AAGGCCCAGTACATGCTCAC	102	XM_0033559855.4
	R: CATAAATGCCCCTCCACCGT		
PepT1	F: CATCGCCATACCCTTCTG	143	NM_214347.1
	R: TTCCCATCCATCGTGACATT		
CycA	F: GCGTCTCCTTCGAGCTGTT	160	NM_214353.1
	R: CCATTATGGCGTGTGAAGTC		

### Western Blotting

Cells were cultured and treated the same as before. After treatments, cells were lysed by 1 × RIPA lysis buffer (Sigma-Aldrich) containing a protease and phosphatase inhibitor cocktail (Thermal Scientific). Cell lysates were centrifuged at 12, 000 g for 10 min at 4°C, and then supernatants were collected. Protein concentration was measured by using a commercial BCA protein assay kit (Thermo Scientific), and bovine serum albumin (BSA) (fraction V) was used to generate the standard curve. For Western blotting, the protein was first denatured in 1 × Laemmli sample buffer containing 5% mercaptoethanol at 95°C for 5 min and 30 μg samples were loaded and separated by electrophoresis in 4–15% Mini-PROTEAN^®^ TGX Stain-Free™ Protein Gels (Bio-Rad). Proteins were then transferred onto nitrocellulose membranes (Bio-Rad). The membranes were firstly blocked with 5% skim milk in tris-buffered saline with 0.1% Tween 20 (TBST) and incubated in specific primary antibodies: rabbit anti-ZO-1 (61-7300, 1:2000, Thermal Scientific) rabbit anti-occludin (71-1500, 1:1000, Thermal Scientific), Phospho- NF-κB p65 (Ser536) monoclonal antibody (MA5-15160, 1:500, Thermal Scientific), NF-κB p65 polyclonal antibody (PA5-16545, 1:200, Thermal Scientific) and beta-actin (β-actin) monoclonal primary antibody (AC-15, 1:5000, Thermo Scientific) that were diluted in 5% skim milk in TBST at 4°C overnight. Membranes were then washed five times with TBST and subsequently probed for 1 h at room temperature with horseradish peroxidase-conjugated goat anti-rabbit IgG (324300, 1:10000, Thermo Scientific) for ZO-1, and OCLN; goat anti-rabbit IgG (324300, 1:2000, Thermo Scientific) for Phospho- NF-κB p65 and NF-κB p65, anti-mouse IgG (65-6120, 1:1000, Thermo Scientific) for β-actin. Then the membranes were visualized using Clarity^TM^ Western ECL Substrate (Bio-Rad) according to the manufacturer’s instruction. The chemiluminescent signal was captured using a ChemiDoc MP imaging system (Bio-Rad), and the intensity of the protein bands was quantified using Image Lab 6.0 software (Bio-Rad). β-actin was used as the internal control to normalize protein determination, and quantitative protein expression levels were presented as relative to the control.

### Barrier Integrity Measurement

Briefly, cells were cultured in 24-well transwell inserts with 0.4 μM pore membrane (Corning Costar) at a density of 7 × 10^4^ cells/cm^2^. TEER value was measured every other day using a Millicell Electrical Resistance System (ESR-2) (Millipore-Sigma) till reaching a stable value (14 days post- differentiation). Cells were pre-treated with capsaicin (100 μM) for 2 h and then stimulated with LPS for 6 h. TEER was measured before and after LPS-stimulation, and the values were expressed as a percentage of initial values before treatments.

Paracellular permeability was measured using 4 kDa FITC-dextran (Sigma-Aldrich). In brief, after LPS-stimulation, 4 kDa FITC-dextran was loaded on the apical side of the insets at a concentration of 1 mg/mL. After 6 h of incubation, medium from the basolateral was collected and fluorescent intensity in the medium was then measured using Synergy H4 hybrid multi-mode microplate reader (BioTek) with excitation and emission wavelengths of 485 nm and 528 nm.

### Immunofluorescent Staining

Cells were cultured onto coverslips, and they were fixed with 4% paraformaldehyde (Pfaffl) after the different treatments, following by the blocking with 5% goat serum for one h at room temperature. And then, cells were incubated with primary antibodies like the anti-rabbit ZO-1 polyclonal antibody (61-7300, 1:100, Thermal Scientific) or Phospho- NF-κB p65 (Ser536) monoclonal antibody (MA5-15160, 1:600, Thermal Scientific) at 4°C overnight. Then, after being washed with PBS three times, cells were incubated with an Alexa Fluor 488 goat anti-rabbit antibody (A32731, 1:1000, Thermal Scientific) for 1 h at room temperature. Cells were rinsed with PBS three times and then were mounted with Vectashield mounting medium with DAPI (Vector Laboratories, Inc.).

For actin staining, fixed cells were rinsed with PBS 3 times, and cells were permeabilized with 0.5% Triton X-100 in PBS at 4°C overnight, following by incubation with Phalloidin, CF488A (94538,1:100, Biotium, Inc., Fremont, USA) at room temperature for 1 h. Rinsed cells were then mounted with Vectashield mounting medium with DAPI, and images were took using a Zeiss fluorescence microscope (Car-Zeiss Ltd., Toronto, ON, Canada).

### Statistical Analysis

Data were analyzed using SAS (the SAS Institute, Cary, NC) and presented as mean ± SEM. The control group was used to normalize data from other groups. Statistical significance was considered at *P* < 0.05. The figures were made using GraphPad Prism 8.12 (GraphPad Software Inc., San Diego, CA).

## Results

### Dose-Effect of Capsaicin on the Viability of IPEC-J2 Cells

**Figure 1:**
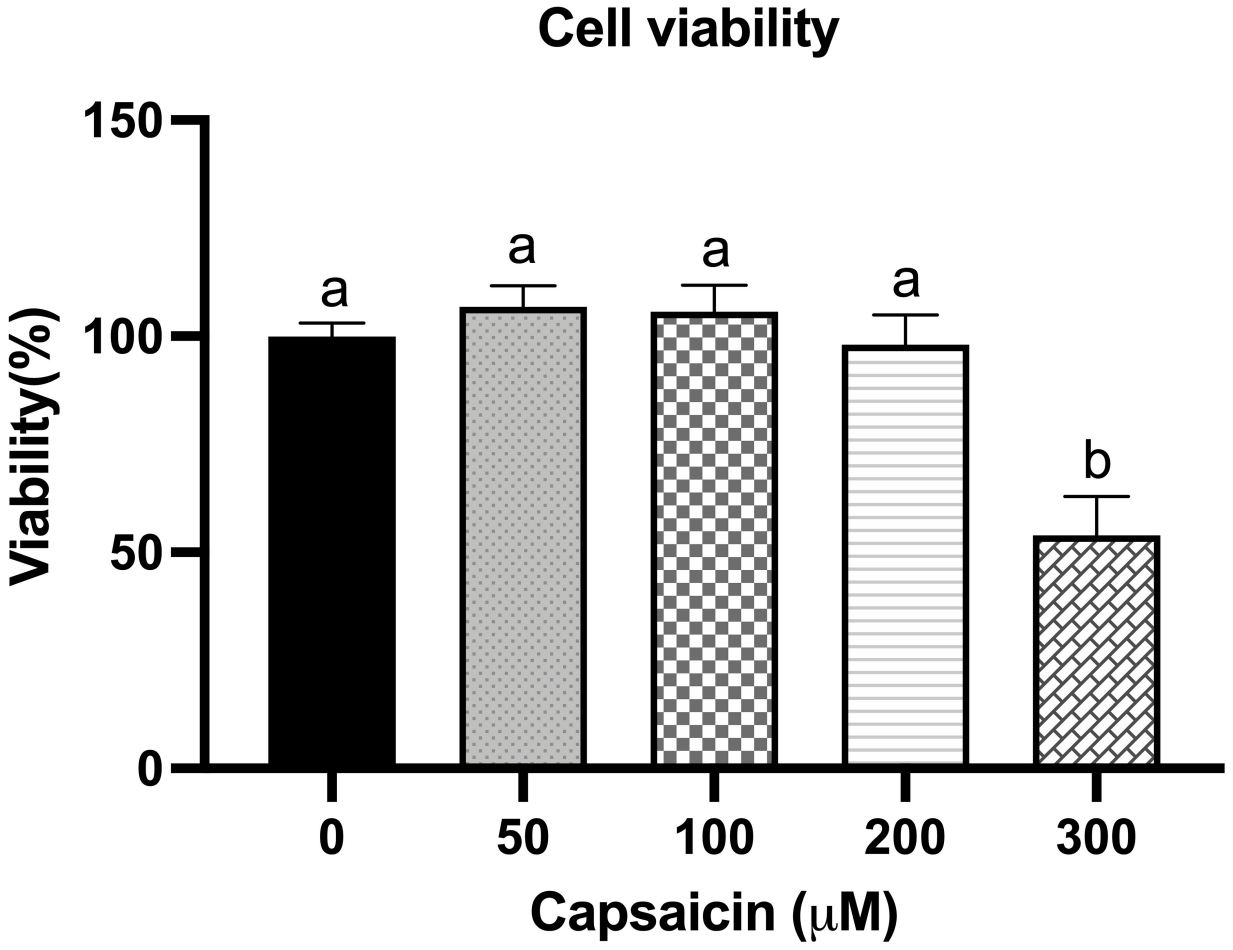
Effect of capsaicin on the viability of IPEC-J2 cells. IPEC-J2 cells were treated with the indicated concentrations of capsaicin for 24 h. Cell viability was measured using the WST-1 assay kit as described in the Materials and Methods. Cell viability data were presented as a percentage of control. Values are presented as mean ± SEM. Different superscript letters on bars (a, b) indicate significant mean differences, *P* < 0.05.

To investigate whether capsaicin is toxic to IPEC-J2 cells, the dose-effect of capsaicin on cell viability was first studied. As shown in Figure 1, cell viability was not significantly affected when the concentration of capsaicin was <200 μM. However, when concentrations were over 200 μM, cell viability decreased significantly compared with the control group (*P* < 0.05). Therefore, 100 μM was used in subsequent experiments.

### Effect of Capsaicin on LPS-Induced Gene Expression and Secretion of Cytokines in IPEC-J2 Cells

**Figure 2:**
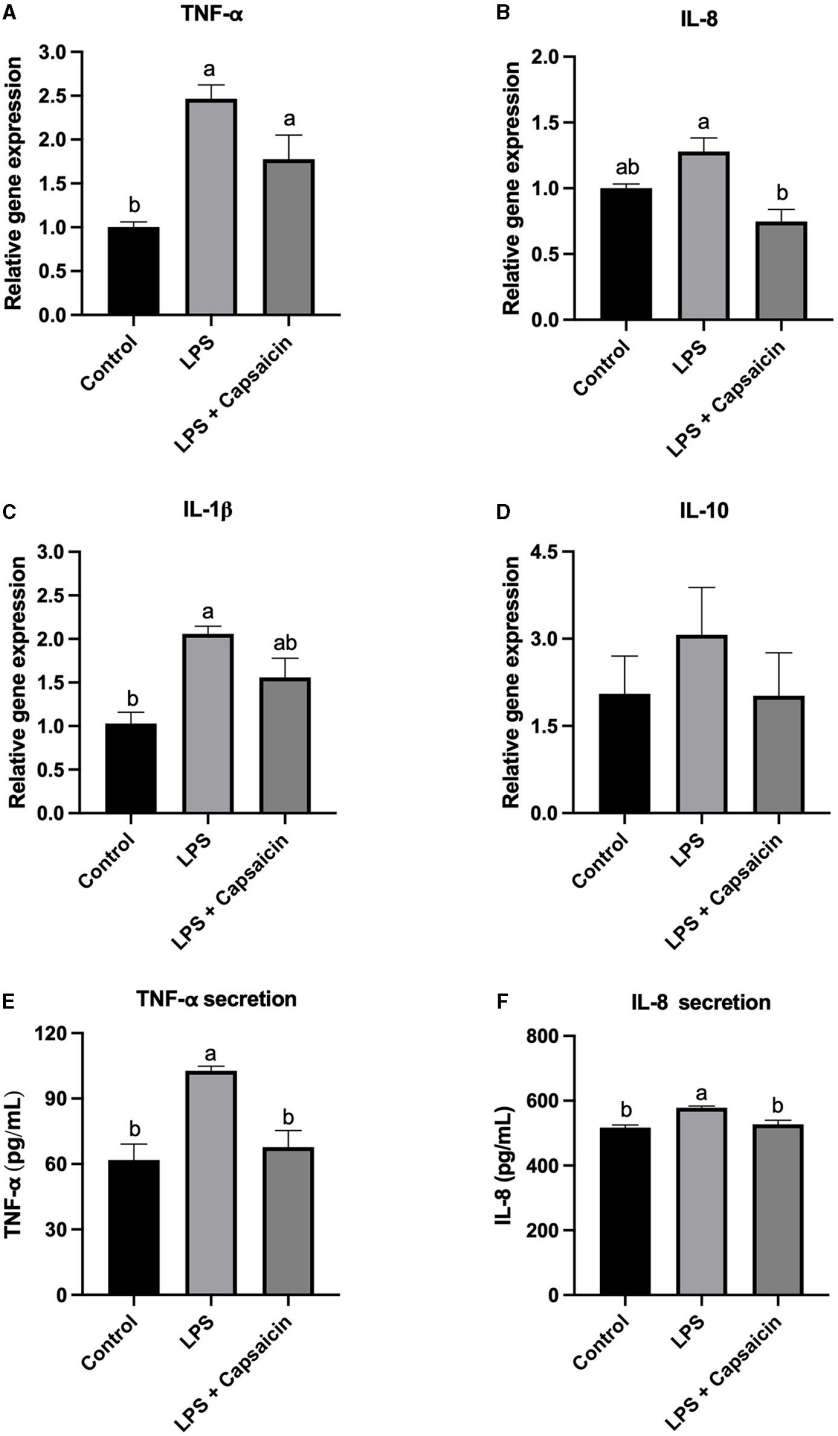
Effect of capsaicin and LPS treatment on gene expression and secretion of cytokines in IPEC-J2 cells. After being pretreated with capsaicin (100 μM) for 2 h, IPEC-J2 cells were stimulated with LPS (10 μg/mL) for 2 h (for real time-PCR assay) or 6 h (for ELISA assay). Total RNA was extracted from cells and the mRNA abundance of TNF-α **(A)**, IL-8 **(B)**, IL-1β **(C)**, IL-10 **(D)**, TNF-α secretion **(E)**, and IL-8 secretion **(F)** was detected by real time-PCR as described in the Materials and Methods. The level of IL-8 and TNF-α in supernatants were measured as described in the Materials and Methods’ Values are presented as mean ± SEM. Different superscript letters on bars (a, b) indicate significant mean differences, *P* < 0.05.

As mentioned previously, cytokines such as IL-1β, IL-8, and TNF-α, as well as IL-10 were selected as inflammation markers, and the effect of pre-treatment of capsaicin on LPS-induced both gene expression and secretion of those cytokines were studied. As shown in Figure 2A, the gene expression of TNF-α in both the LPS treatment group and capsaicin pre-treated group was significantly higher compared with the control group (*P* < 0.05), while there is no significant difference between the LPS treatment group and capsaicin pre-treated group. As for the secretion level of TNF-α, both the control group and capsaicin pre-treated group was significantly lower than in the LPS treatment group (*P* < 0.05), and the difference between the control group and capsaicin pre-treated group was not significant (Figure 2E). Meanwhile, the mRNA abundance of IL-8 in capsaicin pre-treated cells was significantly lower than that in the LPS treatment group (*P* < 0.05), although there was no significant difference between the LPS group and control group (Figure 2B). However, capsaicin pre-treatment did not significantly reduce LPS-induced IL-8 secretion though there was a tendency (Figure 2F). Plus, the mRNA expression level of IL-1β in the control group was significantly lower than in the LPS challenged group (*P* < 0.05), but there was no significant difference between the capsaicin pre-treated group and the control group, as well as the LPS group (Figure 2C). Lastly, there was no significant difference among these three treatment groups at the mRNA transcription level of IL-10 (Figure 2D).

### Effect of Capsaicin on Gene and Protein Expression of TLR4/NF-kB Pathway Associated Genes in IPEC-J2 Cells

**Figure 3:**
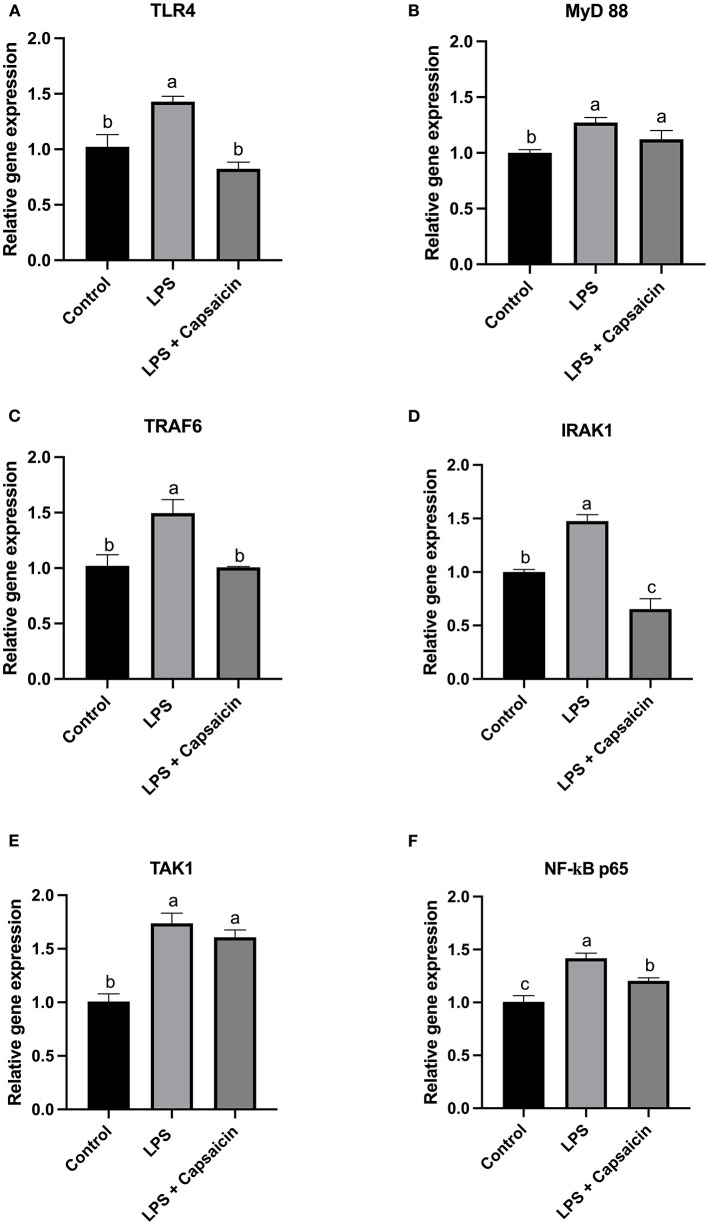
Effect of capsaicin and LPS treatment on gene expression of TLR4/NF-κB pathway associated genes in IPEC-J2 cells. Cells were firstly pretreated with capsaicin (100 μM) for 2 h and then stimulated with LPS (10 μg/mL) for 2 h. Total RNA was extracted and mRNA abundance of TLR4 **(A)**, MyD88 **(B)**, TRAF6 **(C)**, IRAK1 **(D)**, TAK1 **(E)**, and NF-κBp65 **(F)** were determined by real time-PCR as described in the Materials and Methods. Values are presented as mean ± SEM. Different letters on bars (a, b, c) indicate significant differences, *P* < 0.05.

According to results in Figures 3A,C, it showed that the LPS stimulation significantly increased the gene expression level of both TLR4 and TRAF6 compared with both the control group and capsaicin pre-treatment group (*P* < 0.05), but there was no significant difference between the control and capsaicin pre-treatment group. Furthermore, in Figures 3B,E, the mRNA abundance of MyD 88 and TAK1 in both the LPS challenge group and capsaicin pre-treatment group were significantly higher than in the control group (*P* < 0.05), although it didn’t show a significant difference between the LPS treatment group and capsaicin pre-treatment group. As for the gene expression level change of IRAK1 in Figure 3D, it demonstrated that there was a significant difference among these three treatments, and the mRNA level of IRAK1 was the highest in the LPS treatment group, while the lowest level was observed in the capsaicin pre-treatment group (*P* < 0.05). Similarly, the mRNA expression level of NF-κB p65 in the LPS induction group was significantly higher compared with both the control and capsaicin pre-treatment group (*P* < 0.05), but this level was also significantly higher in the capsaicin pre-treatment group than in the control group (*P* < 0.05) (Figure 3F).

**Figure 4:**
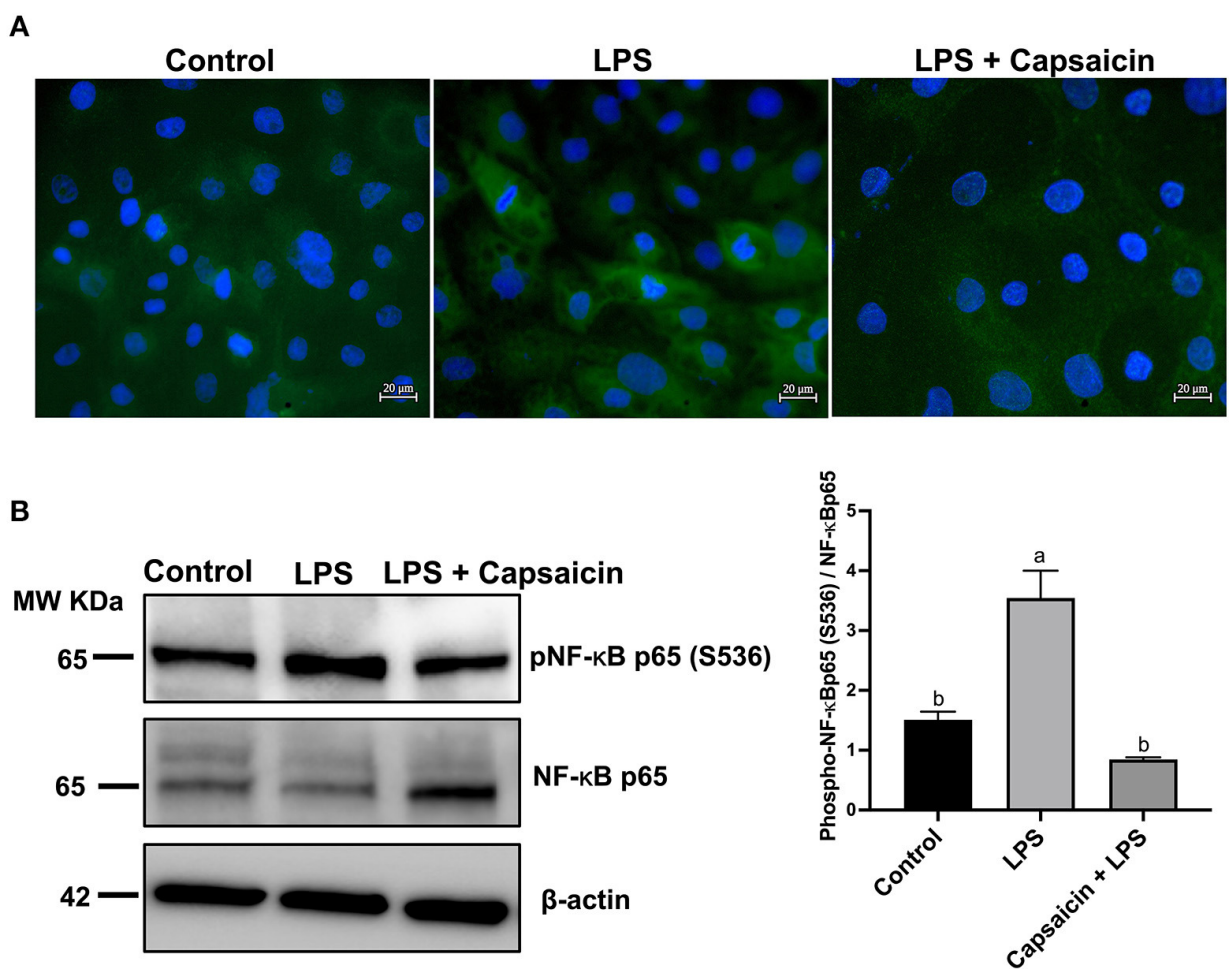
Effects of capsaicin and LPS treatment on the phosphorylation of NF-κB p65 in LPS-challenged IPEC-J2 cells. The level of phosphorylated NF-κB p65 was evaluated using Immunofluorescence staining **(A)** and Western blotting **(B)**, respectively. For Immunofluorescence staining, IPEC-J2 cells were seeded into coverslips at a density of 1×10^5^/well and cultured for 2 weeks. After being pretreated with capsaicin (100 μM), cells were stimulated with LPS. Cells then were then fixed for phosphorylated NF-κB p65 staining as described in the Materials and Methods. As for Western blot, IPEC-J2 cells were cultured in 6-well plates. Cells were firstly pre-treated with capsaicin (100 μM) for 2 h and then stimulated with LPS (10 μg/mL) for 6 h. The protein was extracted and the total NF-κB p65, as well as phosphorylation of NF-κB p65 in LPS-challenged IPEC-J2 cells were detected as described in the Materials and Methods. Values are presented as mean ± SEM. Different letters on bars (a, b) indicate significant differences, *P* < 0.05.

Figure 4A showed that the LPS challenge elicited the activation of NF-κB p65 compared to the control group, while pre-treatment of capsaicin moderately inhibited LPS-mediated NF-κB p65 phosphorylation. Consistently, the results observed in Figure 4A were further confirmed using Western blot. As shown in Figure 4B, phospho-NF-κB p65/total NF-κB p65 densitometry in the cells exposure to LPS for 6 h resulted in a significant increase compared with the control group and pre-treatment of the capsaicin group (*P* < 0.05), but there was no significant difference between the capsaicin pre-treated group and the control group.

### Effect of Capsaicin on Gene and Protein Expression of Tight Junction Proteins in IPEC-J2 Cells

**Figure 5:**
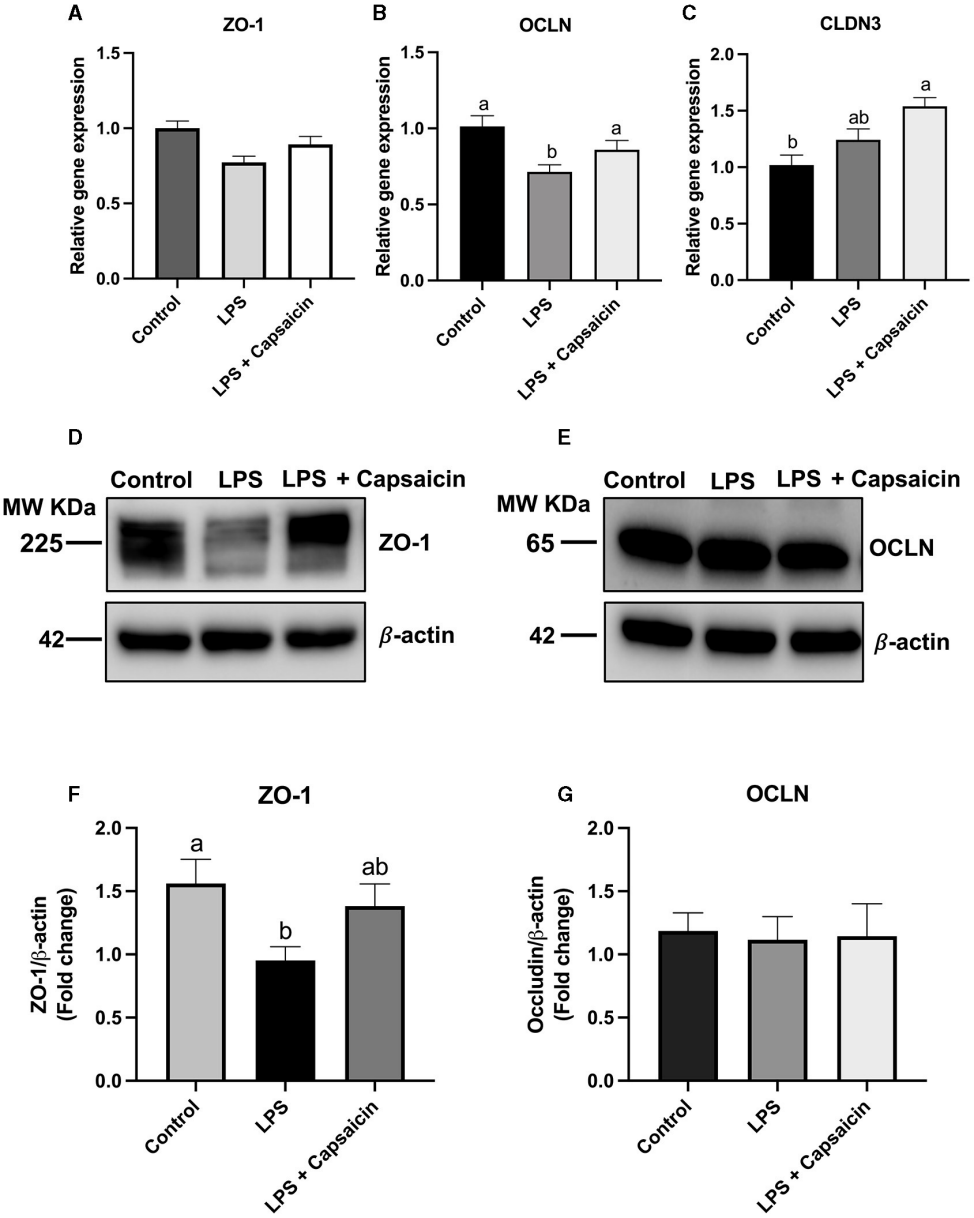
Effects of capsaicin and LPS treatment on both gene and protein expression of tight junction proteins in IPEC-J2 cells. Cells were cultured and treated using the same condition as Figures 2, 4. The mRNA abundance of ZO-1 **(A)**, OCLN **(B)**, CLDN3 **(C)**, protein expression of ZO-1 **(D,F)**, and OCLN **(E,G)** were determined by real time-PCR and Western blotting, respectively. Values are mean ± SEM. Different letters (a, b) on bars indicate significant mean difference, *P* < 0.05.

Figure 5A showed that both LPS and capsaicin had no significant effect on the relative gene expression of ZO-1. Meanwhile, LPS treatment significantly reduced mRNA abundance of OCLN, and pre-treatment of capsaicin had a tendency to recover that change caused by LPS although not significant (Figure 5B). Pre-treatment of capsaicin significantly increased mRNA abundance of CLDN3 compared with that of control (*P* < 0.05) (Figure 5C). In addition, after the stimulation of LPS, the protein expression level of ZO-1 was significantly higher in the LPS treatment group compared with the control group (*P* < 0.05), and the pre-treatment of the capsaicin group showed no significant difference compared with both the control group and LPS stimulation group. Plus, after the exposure to LPS, the ZO-1 protein expression level in IPEC-J2 cells was significantly reduced in the LPS stimulation group (*P* < 0.05), and there was no significant difference between the control group and the pre-treatment of capsaicin group (Figures 5D–G). Neither LPS stimulation nor capsaicin pre-treatment had a significant effect on the protein expression of OCLN (Figures 5D–G).

### Effect of Capsaicin on Morphological Changes in Actin Filaments in LPS-Challenged IPEC-J2 Cells

**Figure 6:**
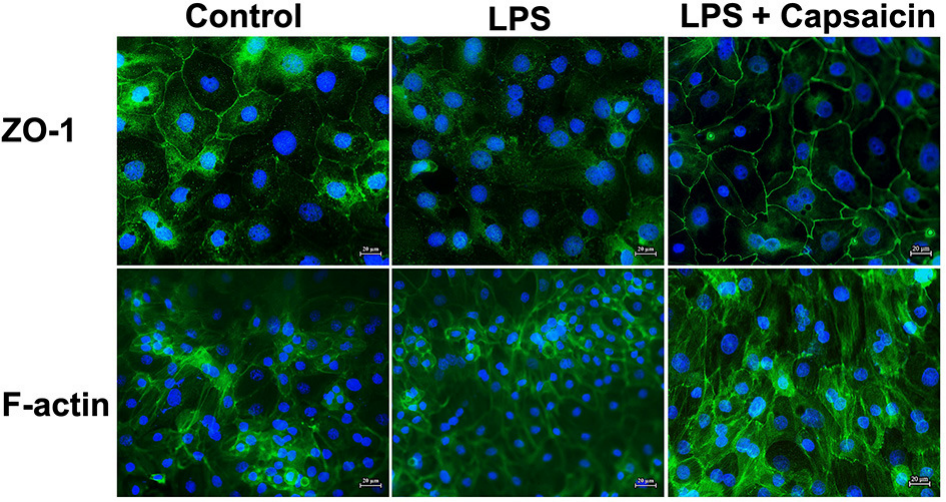
Effect of capsaicin on morphological changes in tight junction protein and actin filaments in LPS-challenged IPEC-J2 cells. Cells were cultured and treated using the same condition as Figure 4. The morphological changes of ZO-1 and actin in LPS-challenged IPEC-J2 cells were detected as described in the Materials and Methods.

As shown in Figure 6, the distribution of ZO-1 protein within the tight junction was damaged in the LPS treatment group compared with the control group. At the same time, pre-treatment of cells with capsaicin redistributed the ZO-1 protein within the tight junction. Meanwhile, LPS-challenged cells showed less density of the filamentous network of F-actin than the control, whereas the pre-treatment of cells with capsaicin alleviated the severity. That suggested that the LPS challenge destabilized the actin filaments of IPEC-J2 cells by reducing the F-actin pool, and the pre-treatment of cells with capsaicin could attenuate the LPS-induced negative effect.

### Effect of Capsaicin on Barrier Function

**Figure 7:**
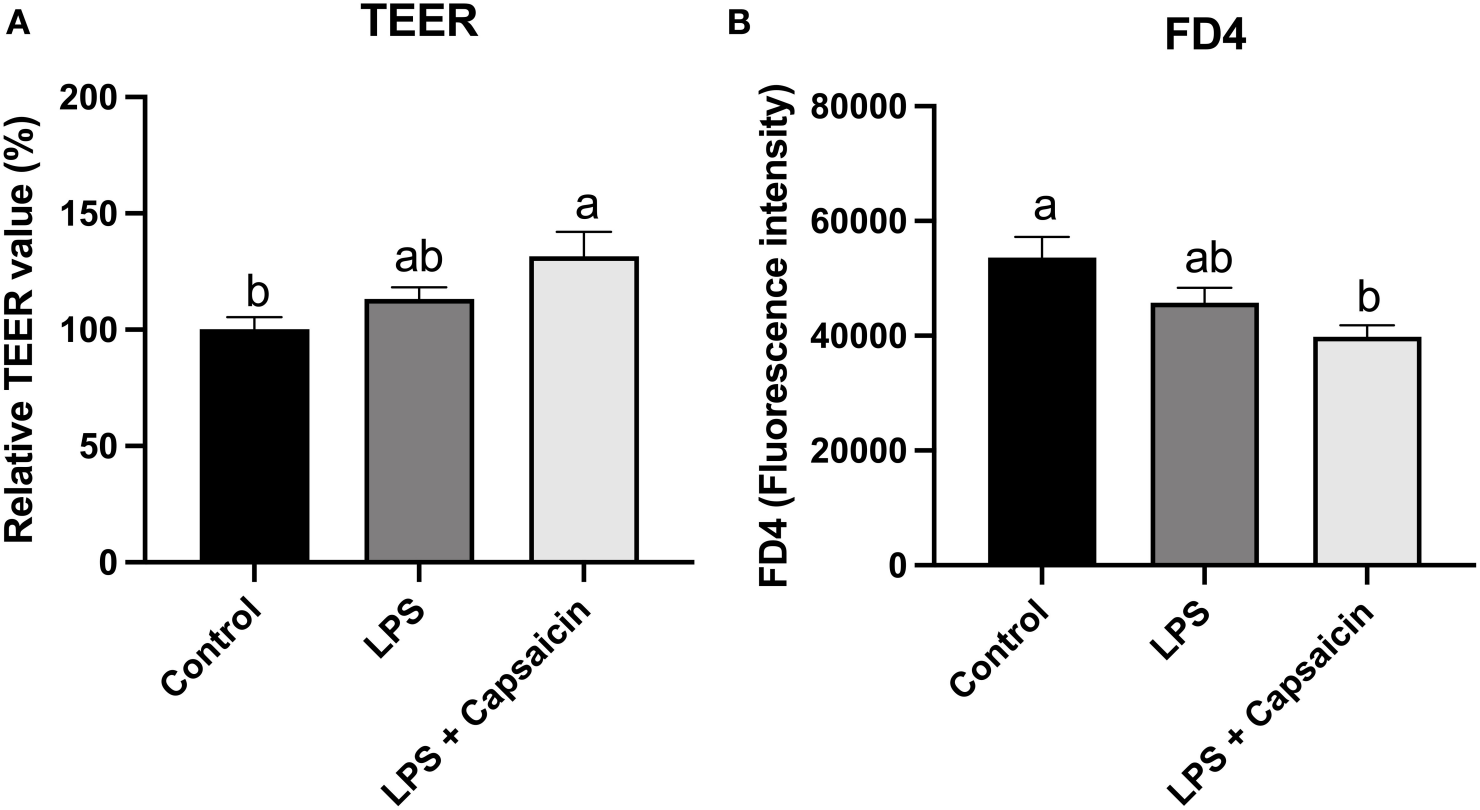
Effect of capsaicin and LPS treatment on IPEC-J2 barrier integrity measured by TEER **(A)** and permeability **(B)**. IPEC-J2 cells were seeded into millicell membrane cell inserts (24-well) and cultured for 2 weeks. Cells were pretreated with capsaicin (100 μM) for 2 h and then stimulated with LPS (10 μg/mL) for 6 h. TEER was measured before and after LPS-stimulation and permeability was determined by FD4 as described in the Materials and Methods. Values are mean ± SEM. Different superscript letters on bars (a, b) indicate significant mean differences, *P* < 0.05.

Barrier function was accessed by measuring TEER and paracellular permeability of FD4 from the apical side to the basolateral side. As shown in Figure 7, LPS treatment had no significant effect on TEER values compared with control. However, pre-treatment of capsaicin significantly increased the TEER value (*P* < 0.05) compared with the control group’s. Conversely, pretreatment capsaicin significantly decreased the leakage of FITC-dextran compared with the control group (*P* < 0.05).

### Effect of Capsaicin on Transporter Gene Expression in LPS-Challenged IPEC-J2 Cells

**Figure 8:**
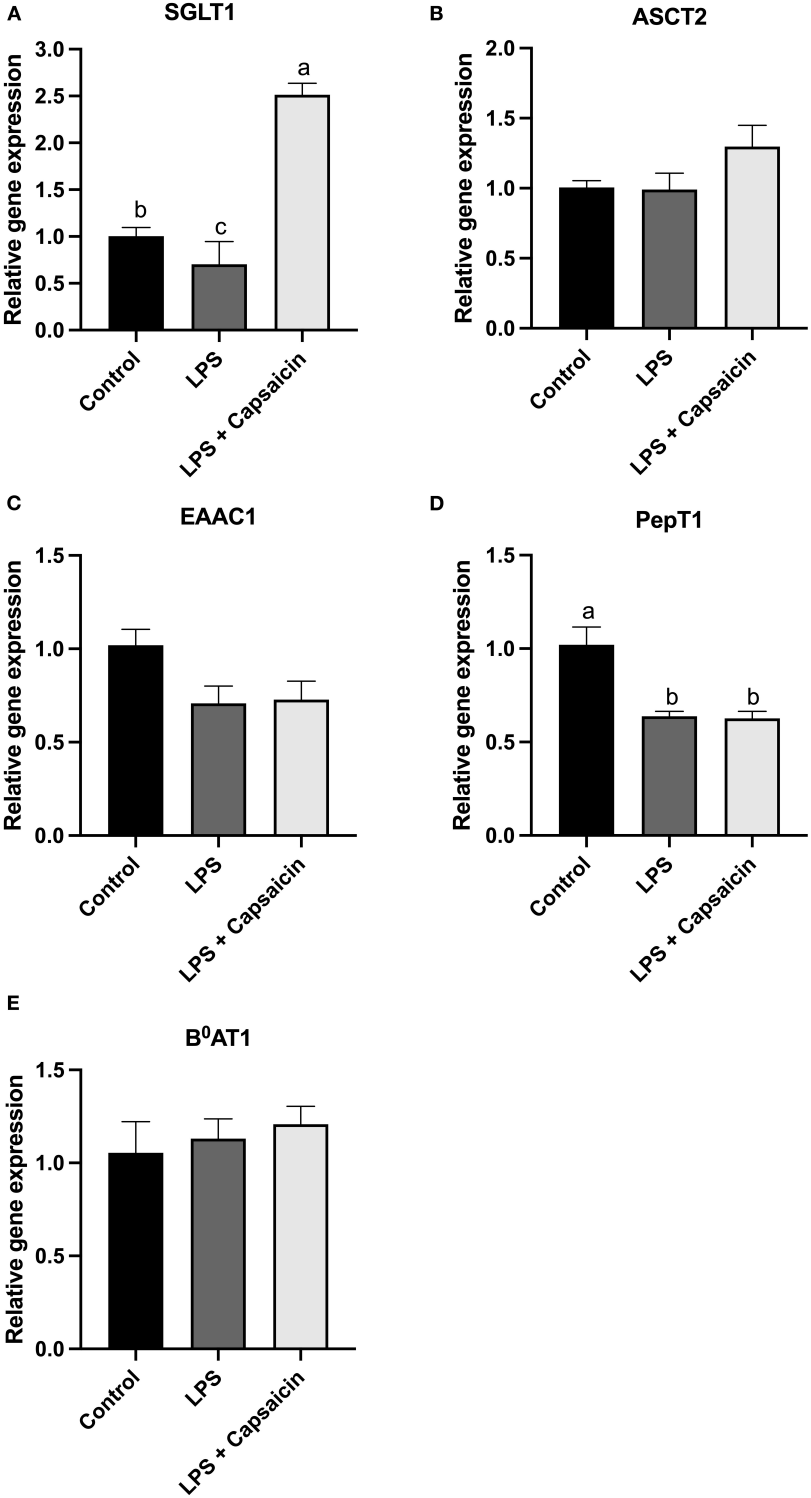
Effect of capsaicin and LPS treatment on nutrient transporter gene expression in IPEC-J2 cells. Cells were cultured and treated using the same condition as Figure 3. The mRNA abundance of SGLT1 **(A)**, ASCT2 **(B)**, EAAC1 **(C)**, PepT1 **(D)**, and B^0^AT1 **(E)** was determined by real time-PCR. Values are mean ± SEM. Different superscript letters on bars (a, b, c) indicate significant mean differences, *P* < 0.05.

As shown in Figures 8A,D, compared with the control group, the mRNA expression level of SGLT-1 and PepT1 were observed to be significantly decreased in the LPS stimulation group (*P* < 0.05). Moreover, the mRNA expression level of SGLT-1 was markedly increased in the capsaicin pre-treatment group when compared with both the control group and the LPS induction group (*P* < 0.05), while the capsaicin pretreatment did not significantly (*P* > 0.05) up-regulated the mRNA expression of PepT1 (*P* > 0.05). Meanwhile, neither LPS treatment nor capsaicin pre-treatment significantly affected mRNA abundance of ASCT2, EAAC-1, and B^0^AT1 (Figures 8B,C,E).

## Discussion

The classic function of the digestive system is to digest and absorb nutrients. The digestive system is also the most important barrier that could keep pathogens or toxins coming from the environment. Since the digestive system has the largest interface between the host and the environment (Flint et al., 2012), this makes the gut the largest organ of the immune system, accounting for 70% percent of the immune system (Zhao et al., 2019). Thus, maintaining a sound intestinal immune system is very important for gut health. Capsaicin is a natural phytochemical present in red peppers, which is widely used as an additive for the daily diet. According to early studies, capsaicin shows several physiological and pharmacological effects (McCarty et al., 2015). Nonetheless, whether it acts as a carcinogen, co-carcinogen, or anti-carcinogen is still controversial, capsaicin has been shown to have potential anti-inflammation, antioxidant, anti-proliferative, and anti-cancer effects (Chang et al., 2011). For instance, researchers reported that capsaicin could relieve inflammation and pain associated with some diseases, including cancer (Thoennissen et al., 2010).

In the present study, the effects of capsaicin on inflammation, barrier function, and nutrient absorption were evaluated using LPS challenged IPEC-J2 cells. Cytokines such as TNF-α, IL-1β, IL-8, and IL-10 were selected as inflammation markers, which have been widely used in the study of LPS-induced inflammatory model in IPEC-J2 cells (Shahzad et al., 2010). Our results demonstrated that the production level of TNF-α in the capsaicin pretreatment group was significantly lower than in the LPS challenge group, and the pretreatment of capsaicin in LPS-challenged cells led to a significant decrease of IL-8 mRNA abundance as well as the tendency to reduce IL-8 secretion. Consistently, the early study also documented that capsaicin could decrease the level of inflammatory cytokines induced by LPS such as IL-1β, IL-6, and TNF-α in human THP-1 cells in a time-and dose-dependent manner (Tang et al., 2015). Another study also reported that pretreatment of capsaicin (100 μM) significantly inhibited both the IL-8 mRNA expression and IL-8 production in *Helicobacter pylori*-infected gastric epithelial cells (Lee et al., 2007). Plus, capsaicin has also been shown to be able to attenuate macrophage inflammatory protein 1 and IL-8 production in human leukemia cells and during discrete stages of sepsis in rats (Choi et al., 2011). All these results demonstrated that capsaicin could attenuate the pro-inflammatory cytokines secretions resulted from the inflammation. According to early studies, weaning was associated with an intestinal transient inflammation in weaned piglets. It was documented that there was intestinal upregulation of inflammatory cytokines like IL-1β, IL-6, IL-8, and TNF-α at weaning (Pié et al., 2004). Moreover, studies have shown that the increased expression of inflammatory cytokines such as TNF-α may develop post-weaning diarrhea (JaimeParra et al., 2013). Therefore, our study implies that capsaicin with anti-inflammation property, has the potential to serves as a diet additive to against inflammation for weaned piglets.

To further explore the underlying mechanism of capsaicin-attenuated LPS-induced inflammatory response in IPEC-J2 cells, changes of gene and protein expression of TLR4/NF-κB pathway associated genes were evaluated in the current study. Based on our results, the gene expression level of TLR4 was significantly upregulated after the LPS challenge, and the pretreatment of capsaicin inhibited this effect. Previous studies also showed similar results as they found that LPS significantly increased the expression of TLR4, and the L-arginine treatment attenuated that detrimental impact caused by the LPS-inducement (Qiu et al., 2019). Meanwhile, results obtained in the current study also showed that the LPS stimulation significantly increased the mRNA expression level of MyD 88, TRAF6, IRAK1, TAK1, as well as NF-κB p65. As all these genes are key players in the TLR4/NF-κB signaling pathway, it implies that LPS can trigger the TLR4/NF-κB signaling pathway and then modulate the production of the pro-inflammatory cytokines in IPEC-J2 cells (Akira and Takeda, 2004). Furthermore, recent studies observed similar results as they demonstrated that TLR4/NF-κB signaling pathway associated genes, as well as inflammatory cytokines, were upregulated in IPEC- J2 cells challenged with LPS (Arce et al., 2010; Shi et al., 2019). Taking together results that the pretreatment of capsaicin could inhibit TLR4/NF-κB signaling pathway associated genes caused by the LPS stimulation, our study implies that the prevention of LPS -induced inflammatory responses in IPEC-J2 cells by capsaicin is associated with the inhibition of the TLR4/NF-κB signaling pathway.

Barriers formed by epithelial and endothelial cells in the small intestine play a very pivital role in preventing endotoxin bacteria invasion, unrestricted exchange of materials, and maintaining gut homeostasis (Bhat et al., 2018). Except for stress, poor gut integrity is the most common source of chronic inflammation in pig production, suggesting that maintaining an effective gut barrier is pivotal for gut health (Pluske et al., 2018). The protective effects of barriers rely on cell-to-cell lateral junction complexes which contain tight junctions, adherens junctions, and desmosomes (Luscinskas et al., 2015). Tight junction proteins include transmembrane proteins like claudins and occludins, cingulin, pals1, and framework forming proteins such as zonula occludens-1 (ZO-1), ZO-2, ZO-3 (Marchiando et al., 2010). All these tight junction proteins play essential roles in maintaining intestinal barrier integrity. The integrity and tightness of barriers are usually associated with the expression of tight junction proteins and evaluated by measuring trans-epithelial electrical resistance (TEER) and permeability by the FITC-dextran flux assay. In this study, we observed that LPS stimulation for 6 h did not cause significant damage to epithelial integrity. This was consistent with one of the previous studies, which reported that 12 h of LPS stimulation did not significantly decrease the TEER and increase the paracellular permeability (Kang et al., 2004). The reason may be complicated as many factors such as the origin and dosage of LPS, duration of treatment and tightness of barrier, and so on, while all these may dramatically influence the response of cells to LPS. However, the capsaicin pretreatment significantly increased TEER and decreased paracellular permeability compared with the control group, suggesting that capsaicin can improve barrier integrity under the LPS stimulation. This conclusion was partly supported by the results of gene and protein expression of tight junction proteins in LPS and capsaicin treated cells. Based on our results, the capsaicin pretreatment was able to significantly increase gene expression of claudin 3 (CLDN3) and restore the decrease of occludin (OCLN) expression caused by LPS, as well as the protein expression of ZO-1. Meanwhile, both LPS and capsaicin treatment did not significantly affect the protein expression of OCLN. One potential reason why capsaicin is able to improve barrier integrity under the LPS stimulation could be attributed to the anti-inflammation property of capsaicin, given that cytokines have been documented to have participated in the regulation of intestinal barrier integrity (Wan et al., 2019). There is evidence that cytokines lead to the disruption of intestinal barrier integrity, contributing to a further increase in tight junction permeability. The inhibition of cytokine resulted in decreased intestinal tight junction permeability (Al-Sadi et al., 2009; Yang et al., 2013). Plus, previous studies also reported that the capsaicin inducement could alter both OCLN and ZO-1 expression and F-actin organization, thus regulate tight junction barrier function (Cong et al., 2013). Moreover, taken together, the pre-treatment of capsaicin has beneficial effects on mitigating LPS-induced inflammation and likely contributed to the improvement of intestinal integrity, which also could reduce the occurrence of chronic inflammation in swine production.

As the primary organ for the digestion and absorption of major nutrients such as protein and carbohydrates, the small intestine plays many roles in the absorption, digestion, and utilization of nutrients (Braithwaite, 1978). The nutrient absorption was mainly facilitated by nutrient transporters (amino acids transporter, peptide transporters, and glucose transporters), which are distributed aligned on the small intestine. These transporters can be classified into two major families, the solute carrier transporters and ATP-binding cassette transporters (van der Wielen et al., 2014). The gene expression level of nutrients transports such as SGLT1, ASCT2, EAAC1, PepT1, and B^0^AT1 may reflect the efficiency of nutrient absorption (Verrey et al., 2009). In this study, we found that LPS stimulation remarkably decreased the mRNA level of SGLT-1, EAAC1, and PepT1, which suggested that LPS stimulation might have a significant effect on nutrient absorption mediated by these transporters. Previous studies also verified that the LPS-induced inflammation generally led to the downregulation of glucose, fatty acid, and L-amino acid transporter mRNA expression (Ling and Alcorn, 2010). Furthermore, we found that pretreatment of cells with capsaicin could recover the reduced mRNA expression level of SGLT1 resulted from LPS stimulation. It is well-recognized that SGLT1 distributed in the apical cell membrane of the small intestine modulates the transport of glucose and galactose from the lumen into intestinal epithelial cells and the expression level of SGLT1 reflected the efficiency of glucose absorption in the small intestine (Lee et al., 1994; Pochini et al., 2014; Vrhovac et al., 2015; Song et al., 2016). In addition, one early study reported that mRNA expression of SGLT1 in weaned piglets was significantly reduced on days 7 and 10 post-weaning (Ikari et al., 2002). Suppose changes in expression result in corresponding changes in glucose transportation. In that case, our results may suggest that capsaicin could be used as a diet additive to improve glucose absorption, especially when challenged with pathogen infection or weaning stress which resulted in a significant decrease of SGLT1 mRNA expression level. However, these results need to be further validated by measuring protein expression and activity of SGLT1, as well as the *in vivo* study.

## Conclusion

In conclusion, we demonstrated that a low concentration of capsaicin (100 μM) could mitigate the inflammation effects under the LPS stimulation in the epithelial cells through the TLR4/NF-κB signaling pathway without toxic effects and improve the intestinal barrier and glucose absorption. The results imply that capsaicin could be used as a feed additive to improve gut health and nutrient absorption. However, this needs to be further verified by *in vivo* studies.

## Data Availability Statement

The original contributions presented in the study are included in the article/supplementary materials, further inquiries can be directed to the corresponding author/s.

## Author Contributions

CY and XZ conceptualized the experiments. CY acquired funding for the research. CY and CZ supervised the study. XZ and BD generated and analyzed the data with assistance from MF and SL and prepared this manuscript. MF, SL, and CY revised the manuscript. All authors contributed to the manuscript and approved the submitted version.

## Funding

This work was financially supported by the University of Manitoba Start-Up Grant (CY, 46561), Canada Foundation for Innovation (CFI), and Graduate Enhancement of Tri-council Stipends (GETs) Program by the University of Manitoba. MF was an NSERC summer undergraduate student in the Department of Animal Science at the University of Manitoba.

## Conflict of Interest

The authors declare that the research was conducted in the absence of any commercial or financial relationships that could be construed as a potential conflict of interest.

## Publisher’s Note

All claims expressed in this article are solely those of the authors and do not necessarily represent those of their affiliated organizations, or those of the publisher, the editors and the reviewers. Any product that may be evaluated in this article, or claim that may be made by its manufacturer, is not guaranteed or endorsed by the publisher.
